# Comparison of reproductive outcomes after laparoscopic tubal anastomosis using conventional (non-barbed) sutures and barbed sutures

**DOI:** 10.1186/s12893-025-03043-z

**Published:** 2025-07-19

**Authors:** Ceyda Karadag, Burak Karadag

**Affiliations:** Department of Obstetrics and Gynecology, Saglık Bilimleri University Antalya Training and Research Hospital, Antalya, Turkey

**Keywords:** Barbed sutures, Conventional sutures, Laparoscopic tubal anastomosis, Tubal ligation reversal

## Abstract

**Purpose:**

This study aimed to compare reproductive outcomes after laparoscopic tubal anastomosis using conventional (non-barbed) and barbed sutures.

**Methods:**

This retrospective cohort study was conducted at a single center between January 2016 and December 2021. Thirty-nine women undergoing laparoscopic tubal anastomosis were divided into two groups: a non-barbed suture (5 − 0 polyglactin suture) group (16 women), and a barbed suture (5 − 0 unidirectional barbed suture) group (23 women). Demographic data, operation times, reversal operation success rates, pregnancy rates, and other factors were compared between the two groups.

**Results:**

The mean operation time was significantly shorter in the barbed suture group (55.8 ± 7.33 min) than in the non-barbed suture group (108.7 ± 17.27 min) (*p* = 0.001). The overall pregnancy rate was significantly higher in the barbed suture group (87%) than in the non-barbed suture group (56.3%) (*p* = 0.037). The rate of intrauterine pregnancy was also significantly higher in the barbed suture group (*p* = 0.041). The intervals between surgery and pregnancy did not differ significantly between the two groups.

**Conclusion:**

The use of barbed sutures in laparoscopic tubal anastomosis can result in shorter operation times and better reproductive outcomes than the use of conventional sutures.

## What does this study add to the clinical work

In laparoscopic tubal anastomosis performed with conventional sutures, the anastomotic line is not clearly visible, making the procedure quite difficult and resulting in a lower success rate. However, when using barbed sutures for this technique, the success rate of the operation is significantly higher.

## Introduction

The number of women desiring family planning increased from 900 million in 2000 to nearly 1.1 billion in 2020. Among women who wish to avoid pregnancy, 77% use modern contraception methods [1]. Female sterilization is the most common method worldwide, with 23.7% of women relying on female sterilization for contraception in 2019 [2].

After sterilization, some women may wish to have children. In this case, in vitro fertilization (IVF) or tubal anastomosis can be performed. Tubal anastomosis can be performed as open surgery but with the development of minimally invasive surgery, it is mainly performed laparoscopically. However, the long surgery time and the considerable surgical skills required constitute substantial drawbacks of laparoscopic surgery [3].

The greatest challenge in laparoscopic tubal anastomosis is undoubtedly the suturing phase. Typically, the aim is to bring the lumens together by suturing the tube at the 3, 6, 9, and 12 o’clock positions with absorbable sutures. However, after the first suture, the visualization of the tubal lumen becomes particularly difficult because the tubal segments are close together. Using very thin sutures (5 − 0 or 6 − 0) causes additional difficulty.

In 2017, Paul et al. described a technique for laparoscopic tubal anastomosis using barbed sutures [4]. In this technique, after passing barbed sutures through the walls of the tubal lumen at the 3, 6, 9, and 12 o’clock positions, the lumen remains clearly visible, as the sutures are approximated one by one after passing through its walls. The use of barbed sutures instead of conventional sutures significantly reduces the operation time. Moreover, this technique can provide greater visibility during anastomosis, as the lumens do not converge after the first suture, as is the case with conventional sutures.

No previous study has compared barbed sutures with conventional sutures in laparoscopic tubal anastomosis.

The primary objective of this study was to compare reproductive outcomes after laparoscopic tubal anastomosis between the use of conventional (non-barbed) sutures and the use of barbed sutures. The secondary objective was to compare the operation times between the two groups.

## Materials and methods

This was a retrospective cohort study of 39 women undergoing laparoscopic tubal anastomosis at Antalya Training and Research Hospital between January 2016 and December 2021. Between January 2016 and December 2017, 16 women underwent laparoscopic tubal anastomosis with 5 − 0 polyglactin sutures (Vicryl; Ethicon, Somerville, NJ, USA) non-barbed suture group), while between January 2018 and December 2021, 23 women underwent anastomosis with 5 − 0 unidirectional barbed sutures (V-Loc; Covidien, Mansfield, MA, USA) barbed suture group).

Before the operation, all women underwent hysterosalpingography (HSG) for proximal tube evaluation, and a sperm analysis was requested from their male partners. Surgery was not performed on women over the age of 40, those with diminished ovarian reserve, patients with ligation observed in the cornual region on the preoperative HSG, those with medical conditions contraindicating pregnancy, and in cases of azoospermia or severe oligospermia on semen analysis. Patients with incomplete medical records were excluded from the study.

This study was approved by the hospital’s institutional review board (no. 5–1, March 3, 2022) and adhered to the STROBE Statement—Checklist of items that should be included in reports of cohort studies to ensure the transparency and reproducibility of the methodology and findings.

### Surgical technique

The patient was placed in the lithotomy position under general anesthesia. A Rubin ‘s insufflation cannula was inserted for uterine manipulation and chromopertubation. The four trocars were inserted as follows: A 12 mm trocar was placed in the umbilicus for the laparoscope. A 5-mm trocar was placed in the midclavicular line at the umbilicus level, and two were placed 2–3 cm medial to the bilateral anterior superior iliac spine.

In the non-barbed suture group, the four-stitch technique was used for anastomosis. The main steps of the procedure were as follows: Methylene blue dye was injected through the intrauterine cannula to identify the obstructed area, the transaction of the tubal stumps, and the removal of scar tissue. If there was a significant difference in the diameters of the tubes, spatulation was performed prior to the anastomosis procedure to equalize the diameters. Anastomosis of the tube was performed sequentially in one layer, including the mucosal, muscle, and serosal layers of the fallopian tubes, using 5 − 0 polyglactin sutures at the 6, 3, 9, and 12 o’clock positions. Tubal patency was checked by identifying the flow of methylene blue dye through the fimbria after the anastomosis. Finally, the defect in the mesosalpinx was re-approximated using 5 − 0 polyglactin sutures.

**Fig. 1:**
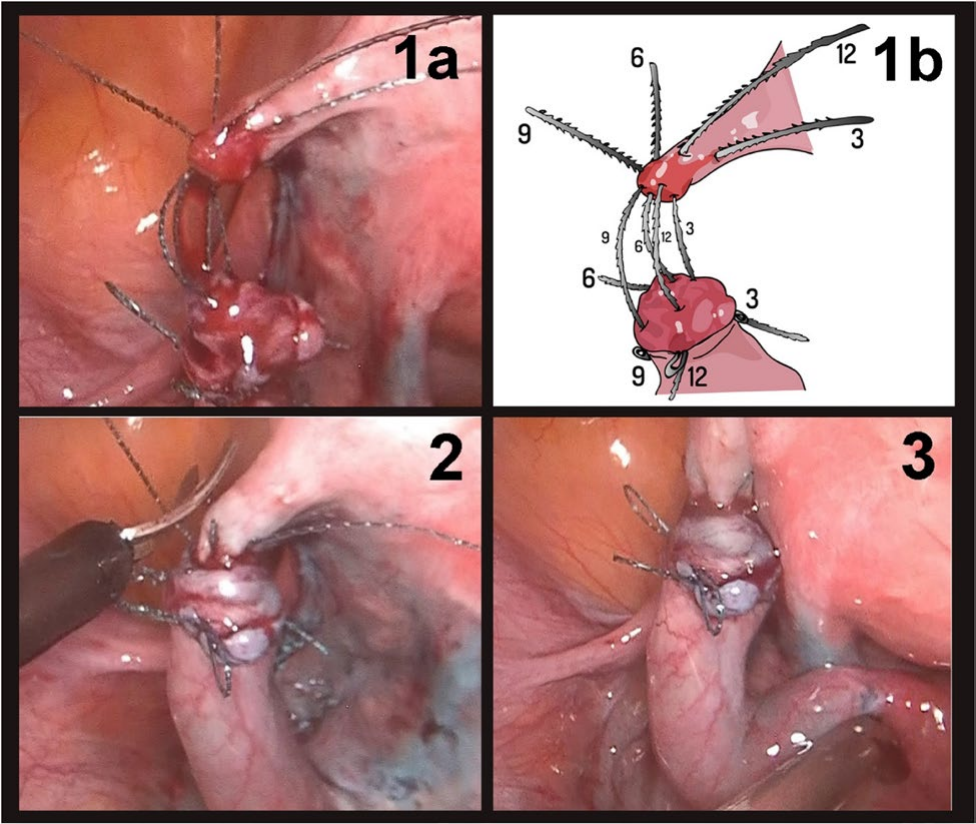
**1a)** A knot was tied to the non-pin part of 5-0 barbed sutures. The sutures were passed at the 6, 3, 9, and 12 o'clock, positions without knotting.**1b)** Schematic representation of the locations of the sutures **2)** After passing through the four quadrants, the tubal segments were brought together by pulling the sutures. **3)** After the anastomosis, the remaining parts of the sutures were cut

In the barbed suture group, all steps up to the suturing stage were performed in a manner similar to that in the non-barbed suture group. A knot was tied to the non-pin part of the 5 − 0 barbed sutures. The sutures were passed through the lumen at the 6, 3, 9, and 12 o’clock positions, without knotting. The tubal segments were then brought together by pulling the sutures, and the mesosalpinx defect was re-approximated using a 5 − 0 polyglactin suture (Fig. 1).

All patients were discharged the following day and were advised to avoid pregnancy for the next two months. HSG was performed three months after surgery to assess tubal patency if pregnancy had not been achieved. Success was defined as the presence of at least one permeable tube. Clinical pregnancy is defined as a pregnancy that lasts 6 weeks (or 42 days) after the onset of the last menstrual period and is confirmed by human chorionic gonadotropin assay.

### Statistical analysis

A post hoc power analysis was performed based on the observed differences in the primary outcome between the two groups, at a significance level of 0.05. This analysis, performed using the “t-tests ◊ Means: Difference between two independent means (two groups)” method in G*Power 3.0.10 indicated 100% statistical power. We controlled for confounding factors by ensuring both groups were comparable in terms of age, BMI, parity, smoking status, sterilization type, and other relevant variables.

The data were analyzed using IBM SPSS Statistics for Windows, Version 23.0 (IBM Corp., Armonk, NY, USA). Continuous variables were expressed as means, medians, and ranges, whereas categorical variables were expressed as percentages and frequencies. The Shapiro-Wilk test was used to assess the equality of variance in the data. Student’s t-test was used for normally distributed data, while the Mann-Whitney U test was used for skewed data. Categorical variables were analyzed using Fisher’s exact test and the chi-square test. A Kaplan-Meier curve was drawn, and the log-rank test was used to compare the probability of conceiving by month. *P*-values of < 0.05 were considered statistically significant.

## Results

A total of 39 patients were followed for a median of 27 months, with a minimum of 3 months and a maximum of 69 months. The demographic and clinical data of the women included in this study are summarized in Table 1. The two groups were similar in terms of age, BMI, and parity (all *p* > 0.05). Similarly, the two groups did not differ significantly in terms of age at sterilization or sterilization technique (*p* = 0.307 and *p* = 0.614, respectively). The mean operation time was significantly shorter in the barbed suture group (55.8 ± 7.33 min) than in the non-barbed suture group (108.7 ± 17.27 min) (*p* = 0.001). The success rates of the tube reversal operation (bilateral or unilateral) were similar in the two groups (*p* = 0.631).

**Table 1:** Demographic and clinical data of the groups

		Barbed group (*n* = 23)	Non-barbed Group (*n* = 16)	*p*
Age (years)		32.6 ± 3.14	31.7 ± 2.74	0.430
BMI (kg/m2)		26.3 ± 2.97	25.8 ± 2.97	0.597
Parity		2 (2–3)	2 (2–3)	0.650
Reason for tubal reversal	New partner	19 (82.6%)	14 (87.5%)	0.281
	Loss of child	3 (13%)	-	
	The desire for a child with a different sex	1 (4.3%)	2 (12.5%)	
Smoking (*n*,%)	Former	6 (26.1%)	2 (12.5%)	0.358
	Current	3 (13%)	5 (31.3%)	
	Never	14 (60.9%)	9 (56.3%)	
Sterilization age (years)		29.5 ± 2.57	28.6 ± 1.96	0,307
Sterilization type (*n*,%)	Pomeroy	16 (69.6%)	11 (68.8%)	0.614
	Coagulation/section	7 (30.4%)	5 (31.2%)	
Type of anastomosis	Ampulla-ampullary	14 (60.9%)	8 (50%)	0.268
	Isthmo-isthmic	5 (21.7%)	7 (43.8%)	
	Isthmo-ampullary	4 (17.4%)	1 (6.3%)	
The time between sterilization-reversal (months)		37.6 ± 22.68	37.5 ± 20.49	0.988
Partner’s age (years)		34.8 ± 4.31	36.1 ± 2.87	0.174
Total antral follicle count		16.5 ± 5.82	15.5 ± 6.13	0.548
Total motile sperm count		99.1 ± 16.7	99.9 ± 24.63	0.484
Operation time (min)		55.8 ± 7.33	108.7 ± 17.27	0.001
Successful reversal of tubes (*n*,%)	Bilaterally	21 (91.3%)	13 (81.3%)	0.631
	Unilaterally	2 (8.7%)	3 (18.7%)	
Tubal length after the reversal (cm)		8.04 ± 1.52	7.93 ± 1.76	0.907
Overall pregnancy (*n*%)		20 (87%)	9 (56,3%)	0.037
Intrauterine pregnancy (*n*%)		19 (82,6%)	8 (50%)	0.041
Extrauterine pregnancy (*n*%)		1 (4,3%)	1 (6,3%)	0.659
The interval from surgery to pregnancy, months		5.8 ± 3.55	7.3 ± 4.06	0.066
Post-operative HSG at three months	Bilateral tubal patency	12 (52.2%)	6 (37.5%)	0.359
	Unilateral tubal patency	4 (17.4%)	4 (25%)	
	Bilateral tubal blockage	2 (8.7%)	4 (25%)	

The overall pregnancy rates were 87% (*n* = 20) in the barbed suture group and 56.3% (*n* = 9) in the non-barbed suture group (*p* = 0.037). The extrauterine pregnancy rates were similar (*p* = 0.659). The intrauterine pregnancy rate was significantly higher in the barbed suture group (*p* = 0.041).

**Fig. 2:**
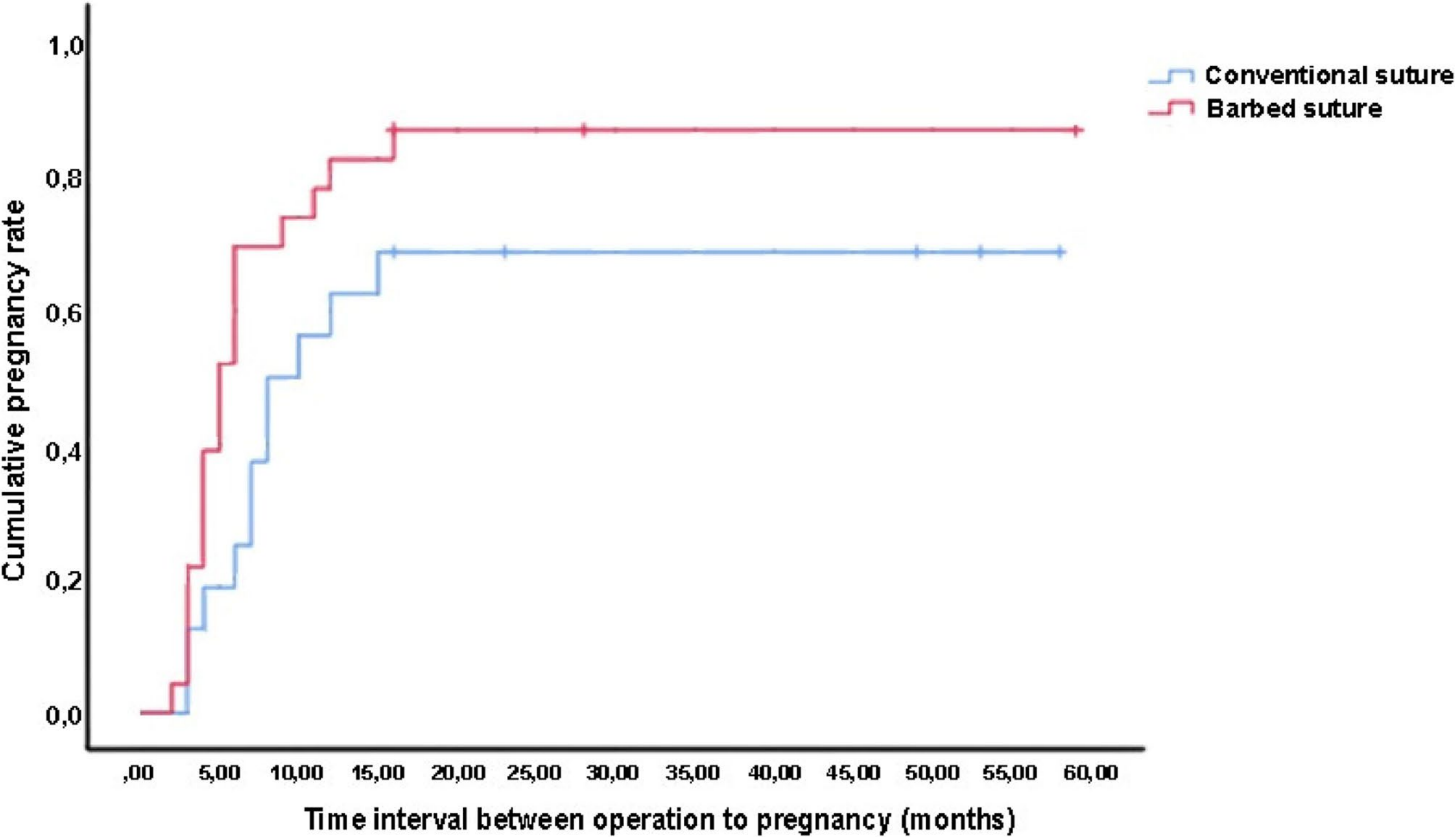
Probability of conceiving by month after tubal anastomosis

The mean intervals from surgery to pregnancy were 5.8 ± 3.55 months in the barbed suture group and 7.3 ± 4.06 months in the non-barbed suture group (*p* = 0.066). The probability of conceiving by month is shown in Fig. 2. The three-month postoperative HSG showed no differences between the two groups in terms of bilateral or unilateral tubal patency in the 3rd month HSG (*p* = 0.359).

## Discussion

This study provides compelling evidence suggesting that the use of barbed sutures offers significant advantages in terms of both surgical efficiency and reproductive outcomes over the use of conventional non-barbed sutures in laparoscopic tubal anastomosis. The use of barbed sutures not only resulted in substantially shorter operation times but also yielded higher overall and intrauterine pregnancy rates.

There are two options for restoring fertility in a woman who has undergone tubal ligation: tubal anastomosis and IVF. The current high success rates of IVF raise the question of whether IVF should be recommended instead of laparoscopic anastomosis. However, a recent review, concluded that tubal anastomosis should be recommended, especially for women under the age of 40 years [5]. The authors also emphasized the need for young residents to be trained in reproductive surgery to ensure appropriate and timely treatment, underlining the continued importance of surgical options in fertility care [5]. These same observations were similarly noted in the review conducted by Garg and colleagues [6]. In the systematic review by Steers et al., it was reported that tubal anastomosis could be more cost-effective than IVF for women under 40, although they did not specify a definitive cut-off age [7]. A recent meta-analysis showed that tubal anastomosis after sterilization is an effective technique for restoring fertility, particularly in women over 35, offering similar or higher success rates compared to IVF [8]. In this study, based on comparative literature on IVF versus tubal anastomosis, we directed patients over 40 years old towards IVF and performed surgery on those under 40.

The tubal anastomosis can be performed using either conventional laparotomy (microsurgical), laparoscopy, or robotic surgery [9]. While the three surgical approaches may result in comparable pregnancy rates, minimally invasive surgery offers significantly greater patient comfort and lower costs [7]. However, laparoscopic tubal anastomosis is complex and requires considerable surgical skills. We know from clinical experience that the use of barbed sutures makes the most challenging part of the operation considerably easier.

The mean operation times for laparoscopic tubal anastomosis reported in the literature range from 108 to 156 min [10–13]. In this study, the mean operation in the non-barbed suture group was 108 min which is consistent with the literature. On the other hand, the mean operation time in the barbed suture group was 55 min which is considerably shorter. This can be attributed to the ease of using barbed sutures, as described by Paul et al. [4]. Barbed sutures offer better visualization of the tubal lumen during the suturing process, which can lead to higher accuracy and efficiency, as the lumens do not converge after the first suture [4]. This has the potential to remove one of the most significant disadvantages of laparoscopic tubal anastomosis, compared to open surgery- namely, the longer operation time [3].

Several factors, including age, male fertility status, tubal ligation method, and postoperative tubal length, affect pregnancy rates after laparoscopic tubal anastomosis. A meta-analysis reported overall postoperative pregnancy rates of 25–83% [6]. In our study, the overall pregnancy rates were 56.3% in the non-barbed suture group and 87% in the barbed suture group. The rates in the non-barbed suture group were similar to those reported in the literature, whereas the rate in the barbed suture group was considerably higher. The higher intrauterine pregnancy rate observed in the barbed suture group could be the result of the more precise anastomosis achieved with barbed sutures. The improved visualization and accuracy provided by the use of barbed sutures can lead to better alignment of the tubal segments, increasing the chance of tubal patency and, ultimately, intrauterine pregnancy. Although the extrauterine pregnancy rates did not differ significantly between the two groups, it is essential to further investigate this outcome, as a higher rate of extrauterine pregnancy may indicate suboptimal anastomosis. In the study by Schippert and colleagues investigating ectopic pregnancy rates after tubal anastomosis, they demonstrated that the surgical technique significantly reduced ectopic pregnancy rates following tubal anastomosis and that precise repair improved fertility outcomes [14].

The intervals between surgery and pregnancy did not differ significantly between the two groups. This suggests that while the use of barbed sutures may result in higher intrauterine pregnancy rates, it may not necessarily shorten the time required to achieve pregnancy. However, this finding should be interpreted with caution due to the small sample size of this study and possible confounders that may have affected the time required to achieve pregnancy, such as age, fertility status, and the presence of other medical conditions.

Despite the retrospective nature and limited sample size of this study, the results are in line with the literature, providing further evidence of the efficacy of laparoscopic tubal anastomosis. Larger, multicenter prospective studies should be conducted to validate our findings and investigate long-term reproductive outcomes. Moreover, comparative studies involving diverse populations and varying degrees of surgical expertise may provide comprehensive insights into the generalizability of our results.

In conclusion, our findings suggest that the use of barbed sutures in laparoscopic tubal anastomosis may result in shorter operation times and better reproductive outcomes than the use of non-barbed sutures. These findings indicate the advantages of using barbed sutures in laparoscopic tubal anastomosis for women who wish to restore fertility after sterilization.

## Data Availability

The data that support the findings of this study are available from the corresponding author, upon reasonable request.

## Abbreviations

BMI: Body Mass Index
HSG: Hysterosalpingography
IVF: In Vitro Fertilization
STROBE: Strengthening the Reporting of Observational Studies in Epidemiology
min: Minutes
HCG: Human Chorionic Gonadotropin
